# “Being useful, I think it's the result of a sick society”: Critical reflections on reproductive politics and markets by women freezing their eggs in Spain

**DOI:** 10.1057/s41292-023-00321-y

**Published:** 2024-03-22

**Authors:** Sara Lafuente-Funes

**Affiliations:** Goethe Universität Frankfurt am Main, Theodor-W.-Adorno-Platz 6, Campus-Westend – PEG-Gebäude, Raum 3.G 072, 60323 Frankfurt am Main, Germany

**Keywords:** Reproductive markets, Egg freezing, Politics of suspension, Cryopreservation, Assisted reproduction, Reproductive biographies

## Abstract

This piece analyzes the way in which women that froze, are considering freezing or are freezing their eggs in Spain think critically about broader reproductive politics in Spain and about assisted reproduction. Drawing partially on previous studies around egg freezing, Thomas Lemke has suggested that cryopreservation practices represent a “politics of suspension” characterized by both reversibility and disposition, and concomitant with broader political inaction (Lemke in Sci Technol Hum Values 48(4):1–27, 2021). Drawing on feminist literature, and on how some of these women think about motherhood, it is relevant to emphasize this ‘suspension of politics’ that takes place along with a “politics of suspension,” meaning that certain matters (such as reproduction and its postponement) are only to be dealt with privately and individually, through marketized fertility preservation programs in this case. Some of the women interviewed describe these programs as useful tools within a problematic context: technologies that give time in a context that leaves them on their own to figure out motherhood (or its absence) in the midst of uncertainty and loneliness. This paper shows their critical views on these matters, while reflecting on how their experiences and desires become increasingly imbricated with the fertility industry in the making of their reproductive biographies (Perler and Schurr in Body Soc 27(3): 3–27, 2021).

## Introduction: Suspending eggs in the midst of a suspension of (family) policies and politics

This paper aims at presenting critical reflections around the role of family-making, motherhood, and assisted reproduction for a generation of Spanish women for whom reproductive choice and decision-making is shifting and is strongly influenced by the reproductive market. I present some critical reflections around reproduction in Spain, its politics and markets, as expressed by some women freezing their eggs. To these reflections, I add an analysis of how some structural factors (pointed out by these women) are also to be found in the narratives of several other women interviewed for this study, even if they did not always address these factors in the form of a critique. I present here a profile of women freezing their eggs that is slightly different to those presented in previous studies around egg freezing elsewhere. I suggest that it might be interesting to look at this profile closely, in order to understand more fully the reproductive situation in Spain, the role of the reproductive market in the country, and the potential of egg freezing as a tool to deal with shifting reproductive patterns more generally.

### A reproductive crisis in a familist country?

Spain has a low fertility rate of 1.19 accompanied by a late average age of 32 for the birth of a woman´s first child (INE 2021). To situate these numbers, three aspects are important: (1) recent demographic studies show an increasing gap between reproductive desires and realities (INE 2018; Castro et al. 2020), in which women say they wish to have more children and earlier than they do. (2) Growing inequalities affect specifically families and children, which has its extreme reflection in the high poverty levels in households with children, specially affecting those with solo mothers^1^ (di Meglio et al. 2018; Ibarra Roca et al. 2021). Finally, (3) a huge reproductive industry has developed in the country, in which the use of assisted reproductive technologies (ARTs) is highly normalized and an estimated 9.3% of all the newborns come from their use^2^ (Seiz et al. 2023). This context has been described as one of structural infertility (Álvarez and Marre 2021) and here I understand it as part of a broader reproductive crisis. The idea of crisis points here to how quickly the Spanish reproductive model is changing,^3^ and to how, in these times of change, reproductive stories are often surrounded by uncertainties, frustrations, and injustices. These uncertainties, frustrations, and injustices are unevenly distributed across class, gender, age, dis/ability, and racial axes, and they affect and are resisted by both women who have children and those considering the possibility of having them.^4^ All of the above takes place at a time when increasing numbers of young women self-identify as feminist (over 80%, according to the INJUVE survey of 2019 as presented in Clavería, 2021) and when feminist discourses are linked to shifts in women’s understanding of reproduction and motherhood (Inmujer 2019). A context in which, and as already pointed out by Alkorta in the early 2000s, “assisted reproduction techniques [are used] as substitutes for active family policies on the part of the government”^5^ (Alkorta Idiakez 2003: 71).

Even though in comparison to other countries like USA Spain has a strong welfare state, with generalized access to public healthcare and with recognized parental leave, in comparison to other EU countries there is much space for improvement. Indeed, public expenditure on families in Spain is below the OECD average (Díaz Gandasegui et al. 2017). Spain can be seen as part of the Mediterranean or *familist* model, “characterized by universal but insufficient public services” and by giving families “especially women, mothers and grandmothers, great responsibility for childcare, with little support from the State” (*Ibid*.: 158). This model tends to be seen as one in which families cover up for caring needs. The fact families take on that responsibility does not necessarily mean they do not long for more state support. Indeed, this idea has been critically revised, showing how in this context “the political thrust of familism is toward greater pressure for welfare state development” offering “an empirically grounded caution against tendencies toward romanticizing low levels of welfare state development in Southern Europe” (Calzada and Brooks 2013, p. 531). That is, even though families are socially and politically expected to take on the responsibility of care work, the lack of state support for this is viewed negatively. This coincides, as we will see later, with the approach of most of the women interviewed in this study, and particularly so with those this paper focuses on.

This negative view of a lack of state involvement in care from the side of citizens might have pushed governments toward some recent improvements. An example of this is the extension of parental leave to 16 weeks, and the fact that non-gestational parents have been granted the same number of weeks for caring for their children as gestational ones.^6^ Even though parental leave has been improved in this way, active family policies are still lacking (without stipends for children, or without specific measures for mothers on their own, for example, who are left with half the time to care for their children that couples have). The lack of active support for childbearing is especially worrying in the context of transformations of the labor and housing markets^7^ that have worsened the quality of life for women and families. These transformations are linked to postponement of the achievement of milestones normally linked to transition to adulthood,^8^ such as leaving the family home (Leccardi 2005; Moreno 2012). Thus, reproductive patterns are shifting along with other major shifts linked to the precarity of the younger Spanish generations, who have been profoundly affected first by the financial crisis of 2008 and now by the COVID-19 pandemic (Jurado et al. 2021). In that sense, even those who expected to have more stable economic positions (such as the highly educated) sometimes experience instability until well into their thirties and forties or throughout their entire working lives, especially women (Torre 2021; Simón and Clavería, 2021). Indeed, even if unemployment has been slowly decreasing since 2014, the last published report from the Institute of Youth indicated that the employment rate for young workers has not yet returned to pre-2008 levels (Torre 2021, p. 70).^9^ Gender inequality can be noted in the paid labor market, as seen by an overrepresentation of women in non-full-time jobs (affecting 36.2% of women and 25.5% of men) and on temporality, which “affects up to 43.5% of young female workers (36.8% of men)” (*Íbid.*: 74). Furthermore, inequality is also to be found within households regarding care distribution, especially when children arrive (Jurado et al. 2021). In this context, 56% of women surveyed in a recent study thought that having children would affect their professional life, and 45% claimed to have suffered discrimination at job interviews through being asked directly about their willingness or not to have children (Chinchilla et al. 2017, p. 45).

### Pregnancy delay, reproductive markets, and egg freezing

In the abovementioned context, motherhood is increasingly being postponed, and the final number of children is reduced. Going back to Alkorta’s statement, it seems that assisted reproduction has emerged strongly as a way of filling the gaps left by the lack of active family policies and as a response (individualized, medicalized, and privatized) to (mainly delayed) reproductive desires of part of the society. Postponement is here linked to the material aspects just presented as well as with the convergence of different expectations placed on women. In Ana Rivas´s words: “When they are young, the expectation is that they will make the most of the education and training opportunities that exist today for women compared to other times, which ends up being a strong social pressure to postpone motherhood.” (Rivas 2017, p. 293) In this context, there is increasing use of egg freezing, offering a new tool for dealing with these shifting times. These treatments, far from being neutral, have been addressed by feminist critique as having the potential to worsen the stratification of reproduction, by offering to some (those who can afford it) more options while making ARTs in general inaccessible to most. Also through embedding neoliberal and anticipatory logics in fertility management (Martin 2010; van de Wiel 2020; Myers and Martin 2021). In Spanish feminist literature, egg freezing has also been addressed as “a new strategy to control women´s bodies” (García Saiz 2018) that “involves depoliticizing the institution of motherhood” (Rivas 2017, p. 303). Even though the specific nature of egg freezing implies introducing fertility, rather than reproduction, into the realm of the medicalized (Martin 2010), it has many points in common with previous ARTs. Thus studies around other treatments might also help to understand its use, such as those pointing to how objectification and agency can cohabit within reproductive clinics in women´s experiences through “ontological choreographie[s]” (Thompson 2005), an insight that is relevant for this paper.

Previous studies around egg freezing have mostly focused on motivations to freeze eggs in the UK and the USA, with a few others studying Israel and Turkey (e.g., Baldwin 2017; Carroll and Kroløkke 2017; Waldby 2019; Göçmen and Kılıç, 2018; Inhorn et al. 2022). The most common finding, which came as a surprise since it contradicted media discourses around fertility preservation, was that a large number of these women were not freezing due to ‘putting careers first’ but rather because of the lack of a partner with whom to raise their (potential and desired) offspring.^10^ Nonetheless, given many women freezing their eggs are in “prestigious occupations” (Kılıç and Göçmen 2018) and that recent studies show how professional women in the USA see freezing as a strategy to anticipate “maternity discrimination” (Zeno 2020), it might be worth returning to the discussion of the role of (poor) work-life balance in contemporary societies. This might be important, if not in terms of motivations, at least in terms of structural conditions shaping reproductive delay and egg freezing.^11^ To better capture the neoliberal turn in the managing of women´s own fertility that often takes place through egg freezing, Carroll and Kroløkke (2017) have suggested the term “responsible reproductive citizenship” and pointed out that egg freezing has been used to “anticipate coupledom” in addition to infertility. This technology, used frequently to “disentangle romance and reproduction” (Brown and Patrick 2018), makes it possible to suspend the reproductive biological aging of women’s eggs, creating new time windows for them to “extend the present” (Lemke 2021) while finding a better moment to (maybe) reproduce.

This paper picks up the gauntlet from Thomas Lemke’s suggestion to think about egg freezing along other practices of cryopreservation as part of a broader “politics of suspension,” a suggestion through which this Special Issue is being proposed. Lemke, drawing partially from feminist work on egg freezing, defines this politics as linked to reserving time to keep options open (extending the present) and conceiving of organic matter as a “standing-reserve” (through the creation and maintenance of biorepositories) (Lemke 2021). This politics of suspension takes place along with what Donna Haraway, following the novel *2312*, labels “the Great Dithering.” That is, our “contemporary political and social inaction” (*Ibid.*: 14/15) in relation to the current problems we are facing. Lemke suggests that while suspending we are not responding. In this paper, I look at the “politics of suspension” by focusing on how some women freezing their eggs think about sociopolitical (in)action in Spain in relation to reproduction and motherhood – and on the role that egg freezing has for them. Thus, I focus on the ways in which some of these women point to social-political inaction (on the part of governments, society, and men) around reproduction, and on how it affects their *dithering* in relation to having children.

## Methods and materials

This article is based on fieldwork undertaken in Spain between 2019 and 2021. It consisted of eight weeks of participant observation in a reproductive clinic (divided into two periods) and sporadic visits to another clinic, in two major Spanish cities, interviews with 22 professionals working at six fertility centers, and interviews with 19 women linked to so-called social egg freezing in three different Spanish cities. Ruling out medical freezing was a choice to focus specifically on non-medical cases. As the line dividing the two is sometimes slippery, I choose to follow the division made by the clinics, which is also the way the Spanish healthcare system draws the distinction, which covers medical but not social egg freezing.^12^ Access to the interviewees was achieved in two different forms (one through collaboration with one clinic, and the other through a mixed of social media and social networks). The collaboration with the clinic was twofold: for a first period I was there, and women undergoing treatment were introduced to me once they agreed to this and after their doctors had explained the research project to them. When access to the clinic was no longer possible (due to the special circumstances of the pandemic), a collaborative partner from the clinic gave women an information sheet about the project, after which some agreed to be contacted – and then agreed or declined to collaborate further. From the women met through these systems, 10 were finally interviewed. The other 9 interviewees wrote the researcher after finding information online (as shared in social media around the project) or via friends/colleagues or previous interviewees. The aim of combining these two contacting methods was to gain access to a more varied sample.^13^ Interviews and fieldnotes were transcribed and analyzed. Each interview was summarized reconstructing the (partial) reproductive biography (Perler and Schurr 2021) of each woman at the stage of the interview, to then search for common themes that were later on inscribed into codes with the help of qualitative data analysis software. This article draws mainly on the interviews with women freezing their eggs, although the observation of doctor’s appointments informed both the questions and the analysis. All of these observations and interviews took place after obtaining consent from all the parties involved, in accordance with the Ethics Committee of the university in which the research project was approved.^14^

Of the 19 women interviewed, 15 had frozen their eggs, two were in the process of doing so, one was undergoing IVF after receiving information that it was too late for her to freeze, and another one was still in the process of deciding what to do after visiting the clinic. Of the 15 women that froze their eggs, five went back to the clinic for an IVF procedure. Of these, three had children resulting from IVF treatment, one had an unsuccessful IVF and was trying again with the last batch of her eggs, and one other woman had turned to egg donation after IVF had failed with her own previously frozen eggs. Even though all these women understood themselves as professionals and most had higher education, not all of them can easily be described as having “high disposable incomes” (Waldby 2019) such as those participating in most previous studies on egg freezing. Nonetheless, they, and particularly the ones with an articulated critique, do have high levels of cultural (and academic) capital, and most have a comfortable economic situation as compared with other socioeconomic profiles.^15^ They are in a good enough economic position to be able to pay for an expensive reproductive treatment that costs between 2500 and 7000 euros,^16^ even if in some cases they did so with help from their families. Nonetheless, some of them still face economic problems or instabilities that complicate their decisions about having children. It is unclear if the existence of this more critical and economically challenged profile is specific to the Spanish context or not, due to either the practice being more widespread, the general socioeconomic situation in the country, or to a mix of both.

Laura Perler and Carolin Schurr, who have studied egg donation in Mexico, argue “that it is necessary to analyse the ways in which the act of donation is entangled with women’s intimate biographies as well as with the wider cultural, political and economic context in which it takes place” (Perler and Schurr 2021: 20). This paper follows this idea while placing the focus on egg freezing. Indeed, in very different ways than those analyzed when looking at egg donors, these women’s reproductive biographies help us to understand reproductive bioeconomies in Spain. This perspective also helps in understanding their rationales in a broader sense, focusing not only on their use of the technology but also on the ways in which reproduction (and its absence) is meaningful to them, both in individual and social terms. In that respect, this concrete piece focuses on the ways in which some of these women think of motherhood as overwhelming, and on the aspects (policies, relationships, or else) they think would make it easier.

I focus here on how some of the women interviewed think about reproduction and on how some structural aspects shape the postponement of reproduction for them. I therefore focus on two aspects (at times concomitant): on the one hand, on how some of the women interviewed express critical views or reflections about the broader context that affected their decision to freeze or consider freezing their eggs. On the other hand, I point to how some of them point to structural factors (such as economic instability or difficult access to housing) as reasons for postponing reproduction. From the 19 interviewees, at least five can be considered as having quite radical critiques of their broader sociopolitical reproductive context, and another three had critical, albeit less radical, stances toward reproductive markets and the commercialization of reproductive treatments. Some of them approached me through the clinic, others through social media, and others through social networks. Most of them had feelings of ambivalence around their own reproductive decisions when freezing their eggs, as they (especially the ones for which the process worked out fine) feel the technique is useful and gave them peace but is double-edged (considering it either a trap, or part of a ‘system’ that should work otherwise).

I decided to focus this piece on these profiles for two main reasons. First, this type of reasoning has not been analyzed in an extensive manner in previous literature on egg freezing.^17^ Second, the discourses presented by the most critical women seemed of great interest as they pointed to something affecting a larger number of the interviewees, which is reflected in the frequency with which economic issues were discussed during the interviews.

## A useful tool, a sick society? Freezing eggs in a context of perceived difficulties for motherhood

For this study, I interviewed women who had frozen their eggs, or were in the process of doing so, at some point over the last ten years, the period in which egg freezing has fully entered the Spanish reproductive market. The first woman from the sample who froze her eggs did it in 2012, and the last one in 2021. Data from the Spanish Fertility Association cover 2010 to 2020, and the figures show a sharp increase, particularly of non-medical egg freezing, during this period. Indeed, while in 2010, 285 women froze their eggs, in 2019 the number went up to 5405, and it is to be expected that the numbers for 2021 or 2022 will be higher.^18^ If in 2010 more than half of these treatments were done by women undergoing gonadotoxic procedures (such as chemotherapy), in 2020 medical causes represented only 24% of the total treatments. That is, 76% of treatments responded to the so-called social or elective freezing – those we have focused on here. Even if freezing eggs is not yet a widespread practice, it is clear that it is quickly growing and is being used by more women with different profiles. Accordingly, most of the women interviewed froze their eggs in recent years. The other side of the coin, the number of women going back to thaw and use their eggs, is still small but keeps on growing. From one pregnancy in 2010, followed by zero births, data from 2020 show 273 pregnancies and 194 births (SEF 2010, 2020). As will be now presented, the reflections some these women have retrospectively on their decision to freeze eggs once they are mothers are more frequently mixed with observations about the role of motherhood in society more broadly, and not only about reproduction as such.

### A “sick society”? Perceived lack of support for reproducing (earlier)

Angels, a 40-year-old worker from the audiovisual industry, froze her eggs in two rounds, first at 36 and then at 37. She went back to use them a bit later as a solo mother and has a child from those eggs. In her interview, she spoke about freezing and motherhood: about the difficulties of having children earlier in life, and more generally. At one point, she passionately said

Society does not support the processes of motherhood and childbearing at all, but **not at all** [original emphasis], this is becoming increasingly clear to me. Furthermore, with time it [society] is becoming less and less supportive. All the policies that are being implemented are there to favor businessmen and businesswomen (…) everything is done to continue feeding external productivity, outside of what is related to upbringing.

Several interviewees, particularly mothers, shared this idea of society not supporting motherhood, or not as much as they thought it should. This was the case with Teresa, an actor aged 47. She froze her eggs while single, living in a shared flat and with unstable jobs at age 38. She is currently mother of a child from those eggs with her partner, with whom she lives. Talking about the limits of public coverage of ARTs, she jumped into talking about support for motherhood in general:

They^19^ need to decide whether they want children or they don’t. In general, I mean. They either want children or they don’t. Because everything is set up so that we^20^ don’t have any. If we want them, it's because we are… well, wonderful heroines. I think it's heroic [to have a child]. There is very little [help?]… and from the male world, in general, I don't say each father, but in general,… I think this should be more of everyone, children, [it should be] more an issue to face as a society. It happens with everything, yes, but… If we^21^ want children, we have to make it easier.

Teresa points here to the individualization of society in general (“It happens with everything”), but focuses on the way in which she feels (Spanish) society leaves the weight of motherhood on the shoulders of women – without the involvement of further social structures and also without enough involvement of men. The generic “they/we” to which she refers links to political debates that problematize low fertility rates (especially relevant within right-wing pronatalist discourse, but also increasingly found in more left-wing discourses^22^), and her emphasis on how little real help she thinks is given to mothers is what makes her talk about having children as heroic.

Ana, a 36-year-old researcher who had an abortion due to lack of economic stability in her early thirties and had been jumping from short contract to short contract since then, was now thinking about freezing her eggs. She lacked a partner and a stable horizon in her job. She was thinking about freezing to try to find either a partner or a more stable job before trying to get pregnant, even if it meant leaving academia. Analyzing her reproductive biography, the idea of motherhood as something difficult to achieve is clear. Several times in her life she thought about having children, especially with a previous partner, but did not do it because of their unstable working and economic situations. In this context, she reflected on how.

I would like there to be more social policies so that having a child would not be such a difficult project, you know?

But why is having a child seen as such a difficult endeavor? These women feel they need to be ready in several ways to become mothers, and some of these aspects (emotional, relational or linked to particular ideals around parenthood) were similar to those found in the existing literature (Baldwin 2017). Nonetheless, references to economic and material worries such as Ana’s were quite present in the interviews, more than in publications based on previous studies elsewhere. This is especially the case if women are thinking about solo-motherhood, which for most of them is not the first or ideal option, but a real one. For instance, Diana, a journalist aged 37, froze her eggs even though she felt she would be happy to become a mother right away. Asked about why she thought about freezing her eggs, she explained

I would be a mother. I have my reasons why I am not, like concrete things. I would love to be a mother and I think I would be a great one, like that. But it is not the right time. The thing is,… my lease ends next year… and that is what I am most afraid of. If they would grant me,… I mean, I live in a state-subsided flat, I live nicely, I pay little,… I work, I earn money. I am not rich or anything but paying what I pay in rent, 300 euros, I can live well and I could have a child, because what I get, what I pay… I could have a child. But the lease ends next year. In one year I do not know what is going to happen with my life, and I cannot get pregnant now and be out on the streets with a baby a few months old.

Several women mention housing as one of the difficulties linked to having children or achieving economic stability. Indeed, and in reverse, Angels very clearly stated that having a flat of her own in the city where she lives, and a family on whom she could rely economically in case of need even if they lived in another city were two major factors making it possible for her to jump into solo-motherhood:

I am super privileged for having been able to do this. Independently of having or not having a partner. (…) I always insist on this: without this flat, and without my mother, no child, no anything. I had my savings and I have been able to invest them in this child. In becoming a mother. (…) And that has been thanks to having a roof over my head, from which I knew nobody could kick me out. Without that? Impossible.

Reflecting on “not being kicked out” is not surprising in a country in which evictions have been so common since the 2008 crisis. Having a flat (at least partially thanks to family money), is key for this solo mother, even though she misses having her family around to help her with her caring load – she at least could count on them financially. This profile, that of women that have moved from their original cities or towns and do not have their families around to help with a child’s upbringing, is quite common in the sample. Indeed, Soraya, a woman who has a clear desire to have a partner before deciding about motherhood and said that this was the main reason why she had frozen her eggs, reflects on how a friend of hers is opting for solo-motherhood but only because her situation is very different from hers, in the sense that

She has her mom living super close by, her aunts, you know… she is from the city originally, has lived here all of her life, she has an immense network of people and I… I just don’t, so: no. That [not being a solo mother] I am positive about.

Having a flat, being able to care for the children, and being able to maintain one’s job were pressing factors when thinking about reproduction. In that sense, even if most of the women interviewed did not discuss careers as the main reason for freezing, it is interesting that almost all of them took for granted that having children would impact greatly their professional lives, especially if they did it when younger. Julia, a translator aged 38 who was now starting the process of freezing, summarized it with this sentence:

Nowadays, no matter what we want to say, to reconcile being a mother with a professional life while being a woman is very, extremely, difficult.

Similarly to what has been experienced in the USA by professional women, some of these women might be anticipating “maternity discrimination” (Zeno 2020, p. 533) on top of infertility itself. Thus, reconciling their professional lives with motherhood is seen as a difficult quest, and things like not having enough maternity leave (especially as solo mothers) or discrimination when finding a job are frequently brought up during the interviews. In this sense, and going back to Teresa’s quote above, it is interesting to ask what she meant when saying “they” should “make it easier” to have children^23^:

There should be longer maternity leave. Also for partners, for men. I mean… leave more time for the whole family, for being able to be off work… I do not know. Care, more care, girl. I do not know.

According to this narrative, shared by others, making things easier includes a broader involvement of men, but mainly of the state in several ways: more public funding for ARTs and more resources for mothers and families. Interestingly, even though most of these women agreed that there should be public funding for ARTs in general, most thought egg freezing as such should not be part of it, and preferred public funding to be directed to help those trying to have or having children. The idea of improvements in maternity leave comes up again and again, and an imagined Europe in which better conditions exist is present. Haizea, for example, who froze her eggs aged 34 after discovering from a routine gynecologist appointment that her ovarian reserve was low,^24^ says:

It is not like we live in a super welfare state, you know? Here it is not like in Northern Europe in which it almost seems to be mandatory to have kids (…) I think it is like a year of maternity leave, or even two! I guess it depends on the country. And for both the father and the mother, and this is like… wow! And with 100% of your salary! This is unthinkable here (laughs), you know?

Or in Ana’s words:

Maybe this sounds crazy, but… could not it be like public housing? You know? A state-subsidized child.” (…) “In other countries they give, I do not know exactly how it works, but they give you more help once you have a child, they give more resources to solo mothers. I am not super aware of the specific details, I just know that it is more complicated here. The same with the whole life-work balance… in other countries they are way more advanced on this.

Worries around work and family balance are present for most of the women interviewed, although most of them insist they do not want that to interfere in their final decision of having or not having children. Nonetheless, it might be among their doubts on the question of timing, and retrospectively, economic and job instability is sometimes pointed to as a reason for postponement. Ana continues reflecting on the difficulties of combining motherhood with work as follows:

A lot of people do not get pregnant because they ask you [at a job interview] How old are you? Do you have kids? You know? (…) And then they hire a man instead (…) if women wouldn’t be affected in their professional careers, only with that, that would be super nice.

Having children is seen as difficult to reconcile with having a professional life, but not having a professional life (which tends to be equated with not having a job) is seen as incompatible with having a child – be it with or without a partner. This is both due to the need for an income and to a certain view of themselves as professionals.

### A useful tool? Longing for easier paths toward motherhood and the usefulness of egg freezing

In this context, most of these women express a longing for an easier path toward becoming mothers. Some also wish that this path allowed them to have children earlier, and some are ok with having them later. Teresa, from the latter group, explains

We, women, we are in this mess,… We want to work, we want to raise children, we want to… We have to do all the decision making, and we have to do this, but also this other thing… People have a lot of opinions on all that, but nobody helps. (…) We need to try to reach a work-life balance. And when it comes to having children: give people options. Options to do it later? Yes, why not? Everything happens later. Everything has changed.

In a way, this quote could be read as linked to the idea of women “wanting it all,” although felt and expressed in a rather different light, which is shared by many other interviewees. These women feel they are supposed to work, be professionals, and have children, but they find it very difficult to do so. As Laia, a researcher aged 41 who froze when she was 39, said

All of that did not line up for me, I guess it can, eh? I guess it can work for some people… But it has not worked all for me. To grow professionally, to endure the precariousness, to find a partner and to have a child, I haven’t had the space for it all, it has not worked out for me.

The great majority of them reflected during the interview on how difficult it is for women in general to work and raise children on top of finding a partner. Following Teresa’s words, this situation leaves women “in this mess,” in which more than wanting it all, they feel they have to “juggle” to do everything that is expected of (and desired by) them in a very individualized way. It is here that egg freezing pops up as an intermediate solution, or a (different type of) “helping hand.” (Strathern, 1992) Paula, who was 32 at the time of freezing and arrived at the clinic after a diagnosis of low ovarian reserve, explained that egg freezing is

An option. Like having one more option (…) I think it is like when you are playing video games and you want to keep on playing but you cannot because you don’t have the time. You push “pause” and you know tomorrow you can still be playing, but the “pause” is there, even if you want to play again in a year. For me it is like that: to pause something that is pressuring you.

Having the option to use this “pause” button is generally seen as something that gave them peace of mind, or a certain degree of calmness in relation to being able to rethink their options with less time pressure.^25^ It helped them to suspend their reproductive aging and to keep options open, to use Lemke’s (2021) phrasing, something that generally made them feel good. This was true especially for those who had successful treatments – for those who felt they had a “good enough” number of eggs cryopreserved. Most of them would recommend freezing to their friends or to women in similar situations. This could lead one to think that their attitude to postponing reproduction through freezing their eggs was positive. Nonetheless, several of these women had ambiguous feelings about the treatment and the idea itself. Angels, introduced before, explains it like this:

Freezing, as a concept of fertility preservation, obviously beyond the cases where you have to undergo chemotherapy or something like that, a priori it seems to me that it is the result of a society that is a little bit sick. Well, of course it's useful, of course it's useful, it's just that in the end,… it's us who have to deal with the shitty situation. In the end ‘I'll take responsibility for everything’ (laughs), won't I? (…) I'm on my own. Of course, well, ‘as you have set up clinics...’ Well, I don't know. Being useful, you know? I think it's the result of a sick society.

Or Olatz, a researcher aged 34, who said

So suddenly it's in your hands to do this or not, but then, I mean, there is no social responsibility (…). I mean: you are responsible [for it], and the one who has to regulate and responsibly manage your own decisions about your fertility, but it doesn't change anything in the way the labor structure works, nothing about how men have to be involved or not involved in all this...

These last two quotes show a very well-developed critique of the individualization of reproductive responsibility for women. Teresa’s quotes above showed this as well, as did some of the reflections from Ana and Laia. This structured discourse was to be found only in these interviews,^26^ but part of what they point to was shared by several other women. That is, even though freezing gives a sense of calmness for those for whom it worked out fine, and it is seen generally as a useful tool they would recommend, some of the women freezing their eggs look at this ‘tool’ with suspicion or resistance. Indeed, they think its existence does not help to solve broader problems linked to the postponement of reproduction, or that it might be pushing them further.^27^ For some of the women for whom the treatment did not add up, this critique is even sharper. All these inputs are interesting as they connect to some of the earliest feminist critiques of ARTs,^28^ but now coming from women who use the techniques for themselves. This can be seen as an example of how critique and embodiment of neoliberal subjectivity can be entangled, and how ambivalent relations to egg freezing, ARTs, and the remaking of motherhood they facilitate unfold in countries with big reproductive industries.

## Discussion

Egg freezing has entered reproductive markets in the last couple of decades on a large scale, and it has also been addressed in sociological, STS, and feminist studies and debates. The main distinctive feature of treatments of the so-called fertility preservation is that they are directed to women who do not want to reproduce immediately when they enter reproductive clinics but are rather anticipating “infertility” (Martin 2010), “maternity discrimination” (Zeno 2022) or “coupledom” (Carroll and Kroløkke 2017) – all of which were found in this study. Egg freezing increases the number of women being targeted by the reproductive industry (van der Wiel 2020) as consumers.^29^ This fertility treatment is part of a broader shift toward an increase use of cryopreservation practices that “seek to arrest processes of decay and dying” through establishing a new form of “suspended life” that can be seen as exposing organisms to a “new onto-political regime” of suspension (Lemke 2021, p. 10). This new cryopolitical regime expands the focus of biopolitics from the two poles of the individual and the population to a third level focused on the governing of body parts and cells (Veit et al. 2023). In the case of governing eggs, fertility and potential reproduction is what is at stake, and women’s reproductive biographies in times of neoliberal agendas and acceleration are being remade in particular ways (Leccardi 2013; Rivas 2017; Perler and Schurr 2021).

Previous studies showed how some women embrace neoliberal ideologies, such as those of self-investment or asset-making, when narrating their experiences of freezing eggs (Brown and Patrick 2018; Kılıç and Göçmen 2018). The profile of women whose reproductive biographies have been presented here walk a similar path to those presented in earlier studies, although some of them are also critical of the broader reproductive context in which they are choosing this path. The way in which the women introduced here make sense of their use of egg freezing, and their reflections on motherhood and reproduction more broadly are informative of a particular moment in Spain. A country in which the role of women has changed rapidly since the end of the dictatorship in the late 70s, right at the same time that Louise Brown proved the success of IVF, and one with a huge development of the reproductive industry (Alkorta Idiakez 2003). A country with a strong feminist movement, and strong presence of feminism in the media and in the political agenda (García and Caravantes 2023), but one that at the same time still places a great burden on families (especially mothers and grandmothers) through a (challenged, but still there) Mediterranean or *familist* model (Díaz Gandasegui et al. 2017; Calzada and Brooks 2013). A country with one of the biggest reproductive markets in the world, let alone Europe.

While most of the women interviewed took full responsibility for their postponement of reproduction, they point as well to other factors making it difficult for them to reproduce earlier – or at all. Careers, finding (good or lasting enough) partners, negotiating with men about having children, housing, and so on, were all mentioned, and a feeling of having children as something overwhelming was very common – particularly if they thought of themselves as solo mothers (one of the key reasons for freezing being to avoid that). These women felt they could not easily intervene in those broader social or relational problems stopping them from jumping into motherhood, although they shaped their reproductive biographies, but they did feel able to do something in more individual terms: freeze their eggs. This was because they were in economically privileged enough positions to enter the reproductive market. This coincides with the finding that “reproductive decision-making tends to be delayed in well-off social contexts” (Simón and Clavería 2021, p. 152) in Spain, although this now happens through a medicalized and marketized intervention. Their stories show how reproductive biographies are increasingly being shaped by the fertility industry (Perler and Schurr 2021). As Rivas anticipated in her work around socio-laboral incentives toward egg freezing in Spain, these women seek to safeguard their reproductive capacity for a different moment – wishing it would be a better one, or giving themselves time to reach a clearer idea of how to fulfill their longing to become mothers. In that frame of action (the individual one), they suspend, click the pause button, and tend to find freezing useful and calming. Even if this means somehow entering into “responsible reproductive citizenship” (Carroll and Kroløkke 2017) some of them do not do so naively or in an uncritical manner. They both embrace and resist egg freezing as a tool for reproductive choice. They find it useful – but acknowledge its double edge and are critical of the broader context leading them to freeze. They are aware of the stratification it entails, and some of them recognize themselves as privileged in this context and problematize their own status.

Some of these women say that freezing gives them peace, but does not solve the broader problems they are facing and that in many cases pushed them to the treatment in the first place: being useful, it can be seen as a “result of a sick society.” All of the above is at times lived with frustration, ambivalence and even anger by some of these women. They long for broader sociopolitical action that would change the conditions in which they are currently acting and deciding. Interestingly, they point to areas in which sociopolitical action could be focused: more social policies, better arrangements for maternity and paternity leave, better conditions for solo mothers, more involvement on the part of men, more public funding for ARTs, etc. This is not to be equated with saying they would have reproduced earlier if these things had been guaranteed, but they thought they could have done so or at least they agreed that these should exist.

Even if not expressed with these same words, some of these women see and problematize:

the role of a neoliberal ideology in which the state and collective responses to social problems are largely absent, and individuals are expected to enact productive citizenship by taking responsibility for their own health, financial, social, and reproductive needs through self‐management, risk reduction, calculation, and optimization. (Myers and Martin 2021, p. 12)

Indeed, some of the interviewees seem to agree with Rivas that “egg vitrification reinforces the individualization process of the neoliberal order, whereby the causes of inequality between men and women and the difficulty of reconciling personal/family and work life are transferred from the structures—labor market, public policies, sex/gender system—to the individual, in this case, to women.” (Rivas 2017, p. 304) And that “we need collective responses by governments and employers” (Myers and Martin 2021, p. 6) – although they do not seem to be forming or engaging in any forms of collective action. Resistance is not, in this case, articulated collectively, and therefore, it does not conform to “body politics” as in other domains (Esteban 2004; Guilló Arakistain 2023), but rather keeps itself within the sphere of individual, marketized action (and frustration).

Charis Thompson´s (2005) insights into the ways in which women moving through clinics acquire agency through objectification is key in understanding the ambiguities, reflexivity, and biographies of women freezing their eggs. The use of this treatment creates for some of them a sense of ambiguity, as they do believe broader sociopolitical changes should take place but feel relief on taking steps to have more time to navigate their own difficulties. In that sense a politics of suspension is enacted, and it is directed at both extending their present and keeping their options open and using their frozen eggs as a “standing-reserve” (Lemke 2021). Nonetheless, this use of cryopreservation is not only accompanied by, but at times also critically perceived as, a ‘suspension of politics’ as the space in which action is taken to affect, shape, and improve living conditions that could help them avoid postponing reproduction altogether, or to avoid the dithering around when and how to reproduce. This follows a very similar pattern to that of feminist critique of the topic of egg freezing, although now voiced by some of the users of these techniques themselves (Harwood 2009; Jackson 2017; Browne 2018; Myers and Martin 2021). To understand the presence of these critiques, it might be important to point to the increasing presence of feminist discourses in Spanish society – not so much drawing from academic critique but rather from a strong social movement (Clavería 2021; García and Caravantes 2023).

## Conclusions

In the study this paper draws on, 19 women who arrived in fertility clinics considering freezing their eggs were interviewed. Around half of them fit nicely into previous literature around egg freezing in other countries: professionals with uncritical takes on egg freezing as a tool to “disentangle reproduction from romance” (Brown and Patrick 2018) who were mainly “anticipating coupledom,” following logics of “responsible reproductive citizenship” (Carroll and Kroløkke 2017) or even embracing ideas of self-investment through freezing (Kılıç and Göçmen 2018). I have focused here on looking at a different profile of women (around half of those interviewed), for whom economic or material aspects were more pressing, and for whom sociopolitical ideas and critiques around motherhood were more elaborated or appeared more visible in their narratives. At times they shared some narratives with the ones presented in previous literature, but they were mostly showing a different side of the story. I have made more visible in the presentation of the findings the views of those women with more critical takes on the role reproduction plays in Spanish society, and on the role of reproductive markets within it, as long as they pointed to issues shared by other interviewees. I did so as this critique was more common than expected in the sample, and as their reasoning points to similar ideas in earlier feminist critiques of egg freezing (Jackson 2018; van de Wiel 2020; Myers and Martin 2021) and to analysis linking this with logics around cryopreservation more broadly (Lemke 2021). These women’s reproductive biographies, which are ever more imbricated with reproductive markets as Perler and Schurr (2021) suggest, represent a growing reality in Spain – a context of growing structural infertility (Rivas 2017; Álvarez and Marre 2021) in a country that can be seen as undergoing a reproductive crisis. The critical takes from these women show a certain resistance to the neoliberal subjectivities offered to them, while at the same time engaging with them through the use of egg freezing. Their ditherings, their critiques, and their ambivalences point to other possible routes that could be taken to alleviate or deal with the situation of reproductive crisis or structural infertility in Spain. Overall, even if some are in more precarious situations, these women are well located within Spanish socioeconomic systems. The fact that even women in these privileged positions still face socioeconomic instability, or have the feeling that their lives and jobs are difficult to reconcile with childbearing, points to a fragile and precarious reproductive scenario in the country – in which the most vulnerable are facing increasing poverty levels, as pointed out in the introduction. The mix of the socioeconomic situation (affected by the advancement of neoliberal agendas and the expansion of a particular repromarket) and the shifts in women’s subjectivities and their take on motherhood (which have not been sufficiently followed by corresponding shifts in men, society or the state) might be resulting in this complex scenario. These shifts could be linked to a (ambiguous, undecided) refusal to continue carrying the burden of childbearing in a context of increasing individualization and privatization – a refusal that is not certain, but rather undecided, doubtful. Within this context, these women ‘buy time’ in the “‘rush hour’ of life” (Bowman et al. 2013) – right before they finally opt in or out of motherhood altogether. Just as their feelings and ideas are ambiguous, undecided or dithering, and, for now, suspended in time, in waiting, so too are their eggs, and, at times, these women’s reproductive biographies themselves.

## Data Availability

The participants of this study did not give written consent for their data to be shared publicly, so due to the sensitive nature of the research supporting data is not publicly available.

## Footnotes

1 Eurostat reports point to how “the risk of poverty or social exclusion [is] particularly concentrated among people living in households with children in Romania and Spain” (di Meglio et al. 2018).

2 This figure, as suggested by the authors of the piece cited, might be slightly lower once births to patients that do not reside in the country are deducted, but it is the best estimate to be found in current literature.

3 As Marta Luxán (2006) points out, this change was not preceded by a stable model, and the so-called baby boom was also an exception. Nonetheless, the shift currently taking place is unprecedented, especially if one looks at what moves that shift in terms of subjectivities, motivations and the framing of reproductive choice (and access to means for exercising it).

4 For a more extended version of what I understand by (Spanish) reproductive crisis, see Lafuente Funes 2021.

5 All references from Spanish sources are self-translated.

6 Parental leave is up to 16 weeks for each parent since 2021, with the first 6 weeks being mandatory to be taken together. In case of solo-motherhood, the mother cannot extend her leave longer than those 16 weeks, so their babies are granted half the time with a caring adult allowed for couples. Some of the interviews presented here are from before this was established, and all of them from before its application was normalized. Before that, only mothers had access to the 16 weeks, and when they were part of couples, their partners had 5, 8, and then 12 weeks.

7 Housing has suffered huge transformations in the last few decades in Spain. On top of the financiarization turn in housing, common to other countries, Spain is turning from a “culture of ownership” to what is described as “generation rent” (Fuster, Arundel and Susino 2018). This shift has introduced a huge cultural shift from previously held expectations of what adulthood should look like, linking social, cultural, and economic changes and giving a sense of continuous uncertainty linked to a huge increase in prices and a very strict regulation linked to a high number of evictions and lasting debts.

8 This is a pattern common to other southern European countries, as the references cited indicate.

9 Indeed, only Greece showed a higher unemployment rate within the EU (Torre 2021).

10 This is described in the US context as a “mating gap” (Inhorn 2023), and is seen in the UK in relation to ideals around parenthood, heteronormativity, and family-making (Baldwin 2017).

11 See also the reflections around this in Kılıç and Göçmen (2018), for a broader analysis of how professional and traditional roles might get entangled in reproductive decision-making processes in the area of egg freezing.

12 Only cases linked to a very clear diagnosis (such as cancer or advance endometriosis) tend to be considered “medical” cases. The fact all these women had to pay for their treatment placed them in the realm of “social” freezing, although some of them only did it after advice from a doctor linked to low ovarian reserve.

13 Accessing women (patients, donors, etc.) through private clinics can be seen as problematic, as it is difficult to rule out the possibility of a private companies “selecting” a particular type of patients (for instance, those happy with the experience). Even though the collaboration with this particular clinic was very good and I am very much grateful to their immense help, as a rule I never leave all contacts come from one source that might have interests on one type of result emerging from the research. On top of this, the patients of this particular clinic tended to be already from a concrete background regarding economic status (considered middle to upper class). To add more diversity to the sample, I announced the research project in some public events, social media and asked people from different backgrounds to share the information with people they knew had undergone this treatment. I then interviewed the women who reached out to me and felt willing to collaborate. In addition to this, one person contacted the PI of the research project this investigation is part of and offered her story to us, and therefore I also interviewed her. This paper draws on experiences of women found through the three formats.

14 Goethe Universität Frankfurt, as part of the ERC funded and approved research project CRYOSOCIETIES.

15 They are indeed privileged if compared to many other women in the country, for example migrant mothers (Moreno-Colom and López Roldán, 2018) or, in the same industry, egg donors (Molas 2021). Nonetheless, it might be the case that not only those enjoying economic stability and in a sound financial position are accessing the treatment, contrary to media representation of the topic and to what has been presented in earlier studies (e.g., Waldby 2019).

16 These are the numbers brought up during the interviews; 7000 euros was the highest figure given, in a case linked to having to do the treatment several times.

17 An exception to this would be in Elissa Zeno’s text on professional women and company-sponsored egg freezing, where four women “were critical of the idea that CSEF represents a solution to their fears about motherhood potentially compromising their careers” (Zeno, 2020: p. 537). Also, Michiel De Proost and Gily Coene (2022) talk very interestingly about ambivalent feelings toward egg freezing in their study on Belgium.

18 In accordance with what professionals from clinics explained in personal communications, as these data have not been published yet.

19 This is a very generic “they” that tends to refer to the government, the state, or society in general.

20 “We” (in contrast to “they”) is to be understood here as “women,” “women who have children,” or “mothers”.

21 And here again, this changes, as “we” is meant to refer to the society in general.

22 The main ARTs regulation might be seen as having a pronatalist approach, especially in relation to the difficulty of discarding eggs and embryos. A general pronatalist discourse is frequently used by right-wing parties to attack the right to abortion, and a general pronatalist discourse is used by political and media figures that can be considered both right and left wing.

23 Or not to have them, as she also insists that not having children is a perfectly good option.

24 Some of the women reached the clinic after their gynecologist (who also worked at reproductive clinics) offered them an anti-mullerian hormone (AMH) test. For more on this type of examination, see Van de Wiel 2020: pp.46–52.

25 This is not always the case, and tends to vary in relation to the age at which they froze and to the result of the extraction.

26 The women with a more structured discourse were mostly working in academia or in the arts. Maybe greater cultural capital, intellectual circles or a closer relation to feminist discourses helped to shape their discourses. Again, researchers and artists came through both social networks and clinical ones.

27 This is particularly the case in relation to men. Two of the women interviewed were worried that the fact they had frozen their eggs made it easier for their male partners or for men in general to ‘keep on pushing’ reproduction back until later in life.

28 Here, I aim at referencing some of the early critiques from the feminist movements in different parts of the world. Even in some of the first documents from FINRRAGE, these ideas were included (among many others), and some were also to be found in the Spanish feminist movement of the 90 s through a publication called “GenCrítica.” Looking at the books by Sarah Franklin (1997; 2013) or Charis Thompson (2005), one can find these earlier critiques explained in detail (along with the authors’ own scholarly developed ones arguments).

29 As with the participation of surrogates and egg providers, the industry has always approached women who did not want to become mothers.

## References

[CR1] Alkorta Idiakez, I. 2003. Los derechos reproductivos de las españolas. En especial, las técnicas de reproducción asistida. *DS: Derecho y Salud* 11 (2): 165–178.

[CR2] Álvarez, B. and D. Marre. 2021. Motherhood in Spain: From the “Baby Boom” to “Structural Infertility”. Medical Anthropology. First Online. 10.1080/01459740.2021.1961246 34372733

[CR3] Baldwin, K. 2017. ‘I Suppose I Think to Myself, That’s the Best Way to Be a Mother’: How Ideologies of Parenthood Shape Women’s Use of Social Egg Freezing Technology. *Sociological Research Online* 22 (2): 20–34.

[CR4] Baldwin, K. 2018. Conceptualising women’s motivations for social egg freezing and experience of reproductive delay. *Sociology of Health & Illness* 40 (5): 859–873. 29602235 10.1111/1467-9566.12728

[CR5] Bowman, D., E. Bodsworth, and J.O. Zinn. 2013. Gender Inequalities and Risk in the ‘rush hour’ of Life. *Social Policy & Society* 12 (2): 277–286.

[CR6] Braun, V., R. Liburkina, and S. Lafuente-Funes. 2023. Die Ordnung der Zellen. Biopolotische Notizen aus dem Labor. In Hoppe,K., J. Rüppel, F. von Verschuer and T. H. Voigt (Eds.) Leben Regieren: Natur, Technologie und Gesellschaft im 21. Jahrhundert. Frankfurt am Main: Campus Verlag.

[CR7] Brown, E., and M. Patrick. 2018. Time, Anticipation, and the Life Course: Egg Freezing as Temporarily Disentangling Romance and Reproduction. *American Sociological Review* 83 (5): 959–982.

[CR8] Browne, J. 2018. Technology, Fertility, and Public Policy: A Structural Perspective on Egg Freezing and Gender Equality. *Social Politics: International Studies in Gender, State & Society* 25 (2): 149–168.

[CR9] Calzada, I., and C. Brooks. 2013. The Myth of the Mediterranean Familism. *European Societies* 15 (4): 514–534.

[CR10] Carroll, K., and C. Kroløkke. 2017. Freezing for Love: Enacting ‘Responsible’ Reproductive Citizenship through Egg Freezing. *Culture, Health & Sexuality* 19 (4): 1–14. 10.1080/13691058.2017.1404643 29185876

[CR11] Castro, T., T. Martin, J. Cordero, and M. Seiz. 2020. La muy baja fecundidad en España: al brecha entre deseos y realidades reproductivas. In: Economistas sin Fronteras (Eds.) Demografía Cambios en el Modelo Productivo, pp. 8–14.

[CR12] Chinchilla, N., L. Jiménez and M. Grau-Grau. 2017. Maternidad y trayectoria profesional en España: Análisis de las barreras e impulsores para la maternidad de las mujeres españolas. IESE Business School – ORDESA (ST-444). https://media.iese.edu/upload/IESEORDESAlow.pdf

[CR13] Clavería, S. 2021. Las actitudes de la juventud ante la igualdad de género. Informe Juventud en España 2020. INJUVE, Madrid.

[CR46] de Proost, M. and G. Coene. 2022. “It gives me time, but does it give me freedom?”: A contextual understanding of anticipatory decision‑making in social egg freezing. BioSocieties. Published online 31 December.

[CR14] Díaz Gandasegui, V., M. Díaz Gorfinkel, and B. Elizalde-San Miguel. 2017. Caring for Children Under Three Years in Two Different Models of Welfare States: The Cases of Spain and Norway. *Journal of Comparative Family Studies.* 48 (2): 157–173.

[CR15] Esteban, M.L. 2004. *Antropología del cuerpo. Género, itinerarios corporales, identidad y cambio*. Barcelona: Bellaterra.

[CR16] Franklin, S. 1997. *Embodied Progress: A Cultural Approach of Assisted Conception*. London: Routledge.

[CR17] Franklin, S. 2013. *Biological Relatives: IVF, Stem Cells and the Future of Kinship*. Durham, NC: Duke University Press.

[CR18] Fuster, N., R. Arundel, and J. Susino. 2018. From a culture of homeownership to generation rent: Housing discourses of young adults in Spain. *Journal of Youth Studies* 22 (5): 585–603.

[CR19] García Saiz, L. 2018. La congelación de óvulos en el ámbito laboral por causas sociales; nueva estrategia empresarial para controlar el cuerpo de la mujer. *Asparkía* 33: 45–59.

[CR301] García, M. and R. Caravantes. 2023. "“Yo soy por las otras están" Investigación sobre el papel y el impacto del movimiento de mujeres y feminista en el reconocimiento de derechos y el avance de la agenda 2030 en el Estado español" Calala Fondo de Mujeres, Barcelona.

[CR20] Göçmen, İ, and A. Kılıç. 2018. Egg Freezing Experiences of Women in Turkey: From the Social Context to the Narratives of Reproductive Ageing and Empowerment. *European Journal of Women’s Studies* 25 (2): 168–182.

[CR21] Guilló Arakistain, M. 2023. *Sangre y resistencia. Políticas y culturas alternativas de la menstruación*. Barcelona: Bellaterra.

[CR22] Harwood, K. 2009. Egg Freezing: A Breakthrough for Reproductive Autonomy? *Bioethics* 23 (1): 39–46. 18945249 10.1111/j.1467-8519.2008.00680.x

[CR23] Ibarra Roca, R., A. Terán and G. Gonzaléz-Bueno Uribe. 2021. Por una prestación para la crianza. Published by: Plataforma de Infancia - Save the Children - UNICEF España. https://www.unicef.es/sites/unicef.es/files/comunicacion/Por-una-prestacion-para-la-crianza.pdf

[CR24] Inhorn, M.C., D. Birenbaum-Carmeli, R. Yu, and Patrizio,. 2022. Egg Freezing at the End of Romance: A Technology of Hope, Despair, and Repair. *Science, Technology, & Human Values.* 47 (1): 53–84.

[CR25] Inhorn, M. 2023. *Motherhood on Ice: The Mating Gap and Why Women Freeze*. New York: NYU Press.

[CR26] INE. 2018. Encuesta Fecundidad. Año 2018, datos avance. “Casi tres de cada cuatro mujeres desearían tener al menos dos hijos” Notas de Prensa, Instituto Nacional de Estadística. https://www.ine.es/prensa/ef_2018_a.pdf

[CR27] INE. 2021. Instituto Nacional de Estadística: Indicadores demográficos básicos – Indicadores de fecundidad.

[CR28] Inmujer. 2019. *Diagnóstico de las mujeres jóvenes en la España de hoy*. Instituto de las Mujeres para la Igualdad de Oportunidades: Catálogo de publicaciones de la Administración General del Estado.

[CR29] Jackson, E. 2018. The Ambiguities of “social” Egg Freezing and the Challenges of Informed Consent. *BioSocieties* 13: 21–40.

[CR30] Jurado, T., C. Castellanos, I. Fernández I., and A. Fernández. 2021. Coresponsabilidad y conciliación de la vida laboral, personal y familiar en España. Desigualdades y transformaciones después de la COVID-19. UNAF: Unión de asociaciones de familias.

[CR31] Kılıç, A., and İ Göçmen. 2018. Fate, morals and rational calculations: Freezing eggs for non-medical reasons in Turkey. *Social Sciences & Medicine* 203: 19–27. 10.1016/j.socscimed.2018.03.014 29533877

[CR32] Lafuente Funes, S. 2021. *Mercados Reproductivos: Crisis, deseo y desigualdad*. Iruñea-Pamplona: Katakrak liburuak.

[CR33] Lafuente-Funes, S. (2023) The Role of Vitrification in Spanish Reproductive Labs: A Cryo-revolution Led by Strategic Freezing. Science, Technology and Human Values. Online first.

[CR34] Leccardi, C. 2005. Facing Uncertainty. Temporality and Biographies in the New Century. *Young: Nordic Journal of Youth Research* 13 (2): 123–146.

[CR35] Leccardi, C. 2013. Postponing motherhood in today´s high-speed society. Conference presented at ICS-UL Lisbon "Childbearing intentions and postponement in times of uncertainty".

[CR36] Lemke, T. 2021. Welcome to Whenever: Exploring Suspended Life in Cryopreservation Practices. *Science, Technology & Human Values.* 48 (4): 1–27.

[CR37] Luxán Serrano, M. 2006. Cambios generacionales en los procesos de formación familiar: La fecundidad de las generaciones de mujeres y hombres a lo largo del siglo XX. *Vasconia.* 35: 301–332.

[CR38] Martin, L.J. 2010. Anticipating Infertility: Egg Freezing, Genetic Preservation, and Risk. *Gender & Society* 24 (4): 526–545.

[CR39] Di Meglio, E., A. Kaczmarek-Firth, A. Litwinska, and C. Rusu. 2018. *Income and Living Conditions; Quality of Life. Eurostat. Living Conditions in Europe -*, 2018th ed. Luxemburg: Publications Office of the European Union.

[CR40] Molas, A. 2021. Taming egg donors: The production of the egg donation bioeconomy in Spain. Unedited PhD Thesis; Monash University. https://bridges.monash.edu/articles/thesis/TAMING_EGG_DONORS_THE_PRODUCTION_OF_THE_EGG_DONATION_BIOECONOMY_IN_SPAIN/17206244

[CR41] Moreno, A. 2012. The Transition to Adulthood in Spain in a Comparative Perspective. *Young* 20 (1): 19–48.

[CR42] Moreno-Colom, S., and P. López-Roldán. 2018. El impacto de la crisis en las trayectorias laborales de las mujeres inmigrantes en España. *Cuadernos De Relaciones Laborales* 36 (1): 65–87.

[CR43] Myers, C. 2014. Colonizing the (Reproductive) Future: The Discursive Construction of ARTS as Technologies of Self. *Frontiers: A Journal of Women Studies* 35: 1.

[CR44] Myers, C., and L.J. Martin. 2021. Freezing Time? The Sociology of Egg Freezing. *Sociology Compass* 15: E12850.

[CR45] Perler, L., and C. Schurr. 2021. Intimate Lives in the Global Bioeconomy: Reproductive Biographies of Mexican Egg Donors. *Body & Society* 27 (3): 3–27.

[CR47] Rivas, A.M. 2017. Incentivos sociales/laborales a la vitrificación de óvulos: ¿Mayor autonomía de las mujeres?. RJUAM, n° 35, 2017-I, 291–306.

[CR48] SEF. 2010–2020. Informe estadístico de la preservación de la fertilidad. Sociedad Española Fertilidad-Registro SEF. www.registrosef.com.

[CR49] Seiz, M., T. Eremenko and L. Salazar. 2023. Socioeconomic differences in access to and use of Medically Assisted Reproduction (MAR) in a context of increasing childlessness, European Commission, Seville, JRC132097.

[CR50] Simón, P. and S. Clavería. 2021. La emancipación juvenil y la familia. Informe Juventud en España 2020., INJUVE. Madrid.

[CR300] Strathern, M. 1992. Reproducing the Future: Anthropology, Kinship, and the New Reproductive Technologies. Routledge, Chapman & Hall, New York, NY. 224.

[CR51] Thompson, C. 2005. *Making Parents: The Ontological Choreography of Reproductive Technologies*. Cambridge, MA: MIT Press.

[CR52] Torre, M. 2021. La juventud y el empleo. Informe Juventud en España 2020. INJUVE, Madrid.

[CR53] van de Wiel, L. 2020. *Freezing Fertility: Oocyte Cryopreservation and the Gender Politics of Aging*. New York: New York University Press. 33661591

[CR54] Waldby, C. 2019. *The Oocyte Economy*. Durham, NC: Duke University Press.

[CR55] Zeno, E. 2022. Synchronizing the Biological Clock: Managing Professional and Romantic Risk through Company-Sponsored Egg Freezing. *Social Problems* 69: 527–543.

